# Intraperitoneal Parasitic Leiomyoma With Torsion

**DOI:** 10.7759/cureus.115759

**Published:** 2026-09-04

**Authors:** Nimy George, Sandra Johns, Nita Sundar, Laly Jose

**Affiliations:** 1 Radiodiagnosis, Dr. Somervell Memorial Church of South India (CSI) Medical College and Hospital, Thiruvananthapuram, IND

**Keywords:** intraperitoneal, leiomyoma, lesion, parasitic, torsion, tumor, uterus

## Abstract

Extrauterine leiomyomas are rare histologically benign tumors. These usually arise in the pelvis and may develop an auxiliary blood supply and become parasitic. Parasitic leiomyoma occurring in patients without prior abdominal or pelvic surgery is extremely rare, with fewer than 30 reported cases in the literature. We report a rare case of parasitic leiomyoma in a 41-year-old woman with no uterine leiomyoma or history of previous surgery. She presented with recurrent lower abdominal pain of one-month duration, and ultrasound revealed a solid, heterogeneously hypoechoic lesion in the hypogastrium. On MRI, the lesion was intraperitoneal and demonstrated a thrombosed vascular pedicle. Laparotomy showed a solid, intraperitoneal mass, adherent to the omentum and anterior parietal peritoneum. Multiple feeding vessels were seen arising from the greater omentum and mesentery with a tortuous, thrombosed vascular pedicle. Histopathology showed interlacing fascicles and bundles of spindle-shaped smooth muscle fibers with cigar-shaped nuclei, consistent with leiomyoma. Imaging plays a vital role in characterizing and differentiating leiomyomas from other masses and in guiding appropriate surgical intervention.

## Introduction

Leiomyomas are the most common benign uterine tumors, which occasionally show unusual growth patterns or occur in unusual locations. Uncommon leiomyomas include parasitic leiomyomas, diffuse peritoneal leiomyomatosis, retroperitoneal leiomyomas, benign metastasizing leiomyomas, and intravenous leiomyomatosis. Occasionally, leiomyomas become “parasitic” when they adhere to surrounding structures, such as retroperitoneal connective tissue, the broad ligament, or the omentum, thereby developing an auxiliary blood supply and ultimately losing their original attachment to the uterus [1].

Although more than 200 cases of parasitic leiomyoma have been reported since the entity was first described by Steiner in 1939, the majority are iatrogenic [2]. Primary (spontaneous) parasitic leiomyoma occurring in individuals without prior pelvic surgery remains exceptionally rare, with only a limited number of cases reported in the literature [3].

Extrauterine leiomyomas often arise in the genitourinary tract and are most commonly located within the pelvis (vulva, ovaries, urethra, and urinary bladder), although they may occur at other anatomical sites [4]. Owing to their rarity, extra-pelvic leiomyomas may easily be confused with other pelvic or extra-pelvic masses.

Clinically, these lesions can present as extrauterine pelvic masses that exert pressure on the urethra, bladder neck, or ureter, leading to varying degrees of urinary outflow obstruction or resulting in secondary hydroureteronephrosis [5].

Other atypical forms of leiomyoma include benign metastasizing leiomyoma, characterized by multiple well-differentiated leiomyomas at sites remote from the uterus, most commonly the lungs and, rarely, the heart, brain, lymph nodes, bone, and skin. Other rare locations include the sinonasal cavities, orbits, and kidneys.

While the historical medical literature interchangeably describes extrauterine smooth muscle tumors as "parasitic fibroids" or "wandering fibroids," this manuscript strictly uses the formal pathological designation "parasitic leiomyoma" [6]. This approach aligns with the International Federation of Gynecology and Obstetrics subclassification system, which officially categorizes these rare variants as type 8 leiomyomas.

## Case presentation

A 41-year-old woman with a regular menstrual history and primary infertility presented with recurrent mild lower abdominal pain of one month’s duration. She did not have any bowel- or bladder-related symptoms. On examination, a firm, non-tender, smooth mass was palpable in the hypogastrium and umbilical region and had limited vertical movement. The mass was equivalent in size to a 20-week gravid uterus, and a provisional diagnosis of subserous leiomyoma was made. Chest radiograph (Figure 1), electrocardiogram (Figure 2), and complete blood count (Table 1) were normal.

**Figure 1:**
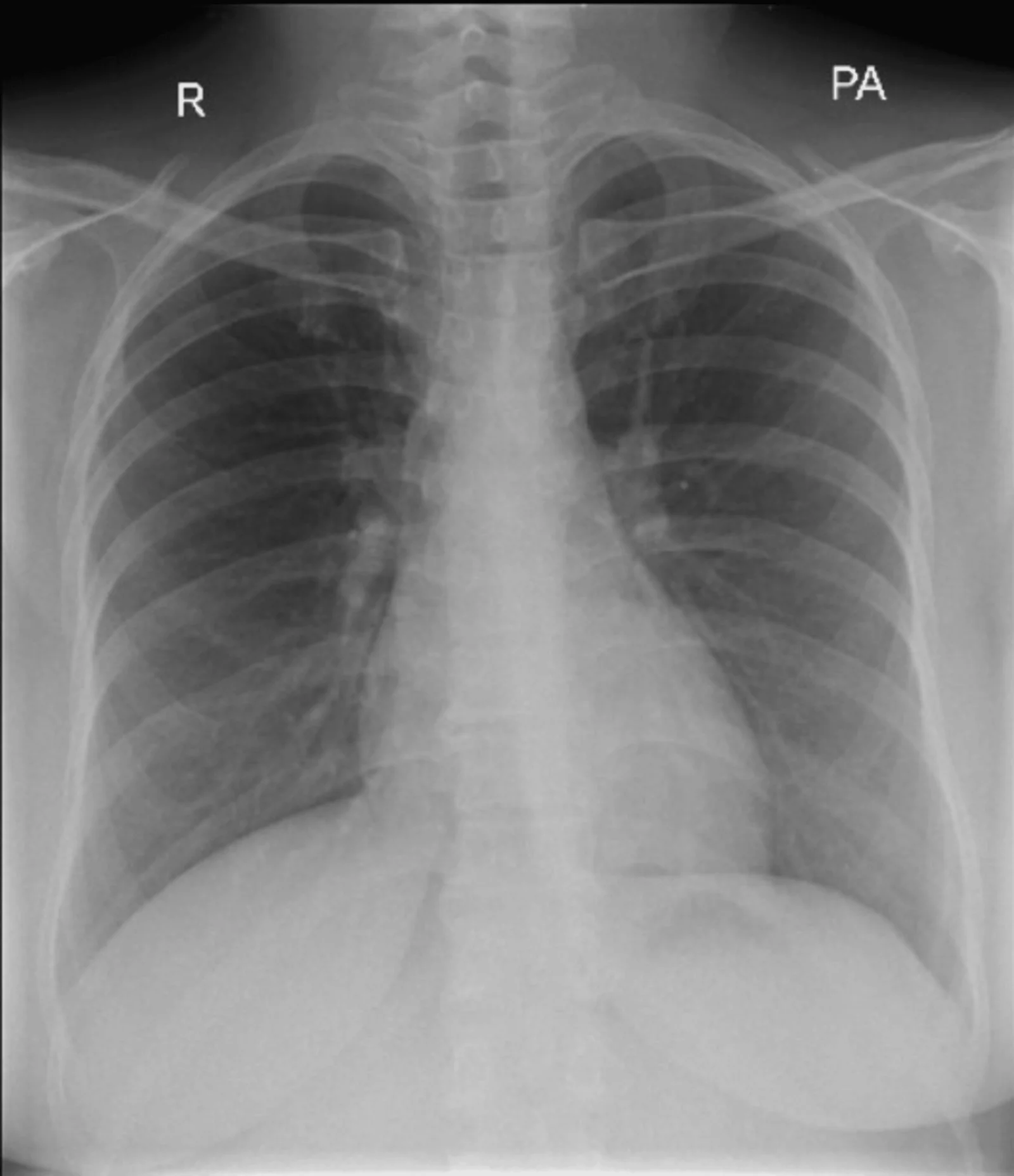
Chest radiograph

**Figure 2:**
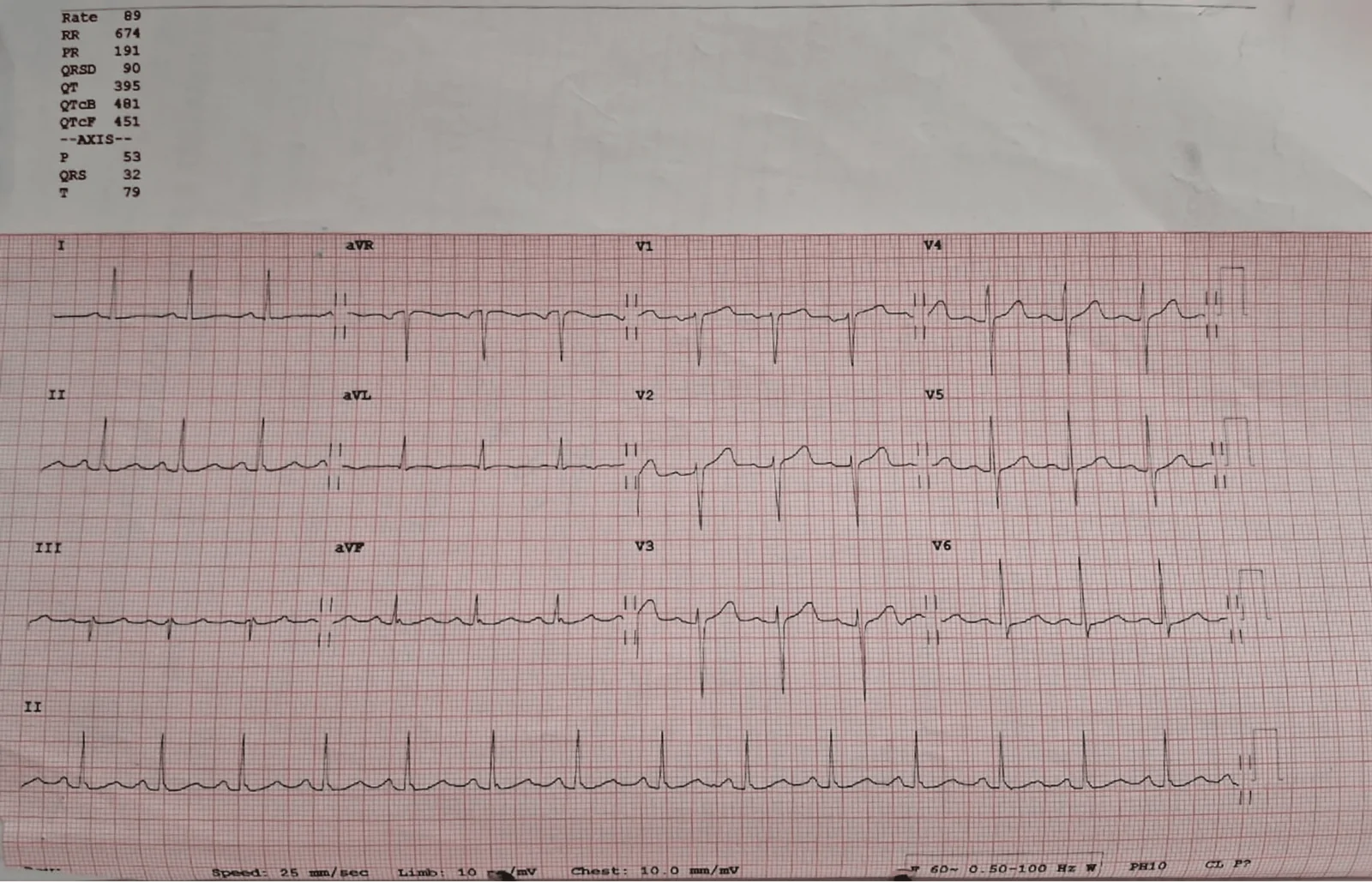
Electrocardiogram

**Table 1:** Laboratory data of the complete blood count MCV: mean cell volume, MCHC: mean cell hemoglobin concentration, RDW: red blood cell distribution width, RBC: red blood cell

Test name	Result	Reference value
Hemoglobin	12.3 g/dL	12.5-16.0 g/dL
Total count	9850 cells/cu.mm	4000-10500 cells/cu.mm
Neutrophil	72%	54-62%
Lymphocyte	24%	25-33%
Eosinophil	3%	1-6%
Monocyte	01%	3-7%
Basophil	0%	0-2%
MCV	83.9 fL	83-101 fL
MCH	27.5 pg	27-32 pg
MCHC	32.8 g/dL	31.5-34.5 g/dL
RDW	14.3%	11.6-14%
RBC count	4.47 million/cu.mm	4-5.2 million/cu.mm
Platelet count	4.6 lakhs/cu.mm	1.5-4 lakhs/cu.mm
Packed cell volume	38%	36-46%

Diagnostic assessment

Ultrasound of the abdomen showed a solid, well-defined, heterogeneously hypoechoic lesion of ~9 cm with a few hyperechoic areas in the hypogastrium, abutting the fundus of the uterus (Figure 3). Both ovaries were normal. Transvaginal ultrasound (TVS) showed a normal uterus with no focal lesions. The hypogastric mass was not visualized on TVS. Hence, a diagnosis of pedunculated subserous leiomyoma was considered, and further evaluation with contrast-enhanced MRI was performed to assess atypical features and blood supply.

**Figure 3:**
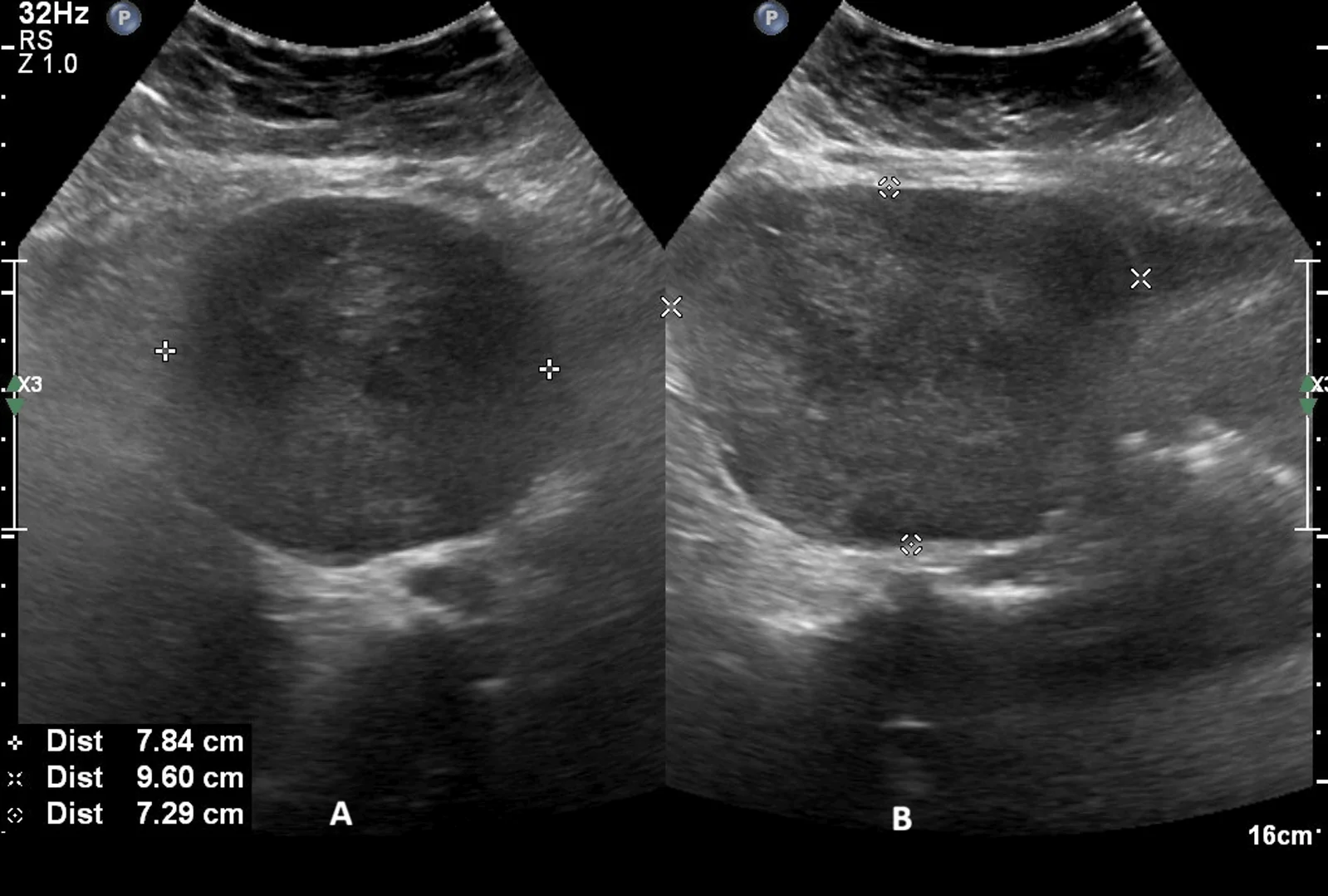
Ultrasound images Sagittal (A) and coronal (B) sections of a well-defined, solid, heterogeneously hypoechoic lesion with a few hyperechoic areas, in the hypogastrium, abutting the fundus of the uterus.

MRI of the pelvis showed a well-defined, solid, lobulated mass, ~9 cm in diameter. The mass was hypointense on T1-weighted imaging and heterogeneously hyperintense on T2-weighted imaging, with a T2-hypointense capsule, in the hypogastrium, more toward the right (Figure 4A-4B). The lesion showed curvilinear areas of restricted diffusion with enhancement corresponding to T2-hyperintense areas (Figure 5A-5D). The lesion was seen separately from the uterus with intervening small-bowel loops (Figure 6A-6B). Serpiginous, tortuous, non-enhancing T1-hyperintense tubular structures were seen in the right posterolateral aspect of the lesion, suggestive of a thrombosed vascular pedicle (Figure 6C).

The lesion was considered an atypical extrauterine smooth muscle tumor based on its well-defined morphology, separation from the uterus, and vascular characteristics. However, the areas of diffusion restriction and heterogeneous T2 signal raised the possibility of other mesenchymal tumors, including mesenteric desmoid tumor, gastrointestinal stromal tumor (GIST), and leiomyosarcoma. A parasitic leiomyoma was considered among the differential diagnoses, particularly given the presence of a thrombosed vascular pedicle and extrauterine feeding vessels. A definitive diagnosis was established following surgical excision and histopathological examination.

The key diagnostic clues in this case included the absence of continuity with the uterus, preservation of both ovaries, a thrombosed vascular pedicle with extrauterine feeding vessels, and imaging evidence of myxoid degeneration. Together, these findings raised suspicion for primary parasitic leiomyoma despite the atypical MRI appearance.

**Figure 4:**
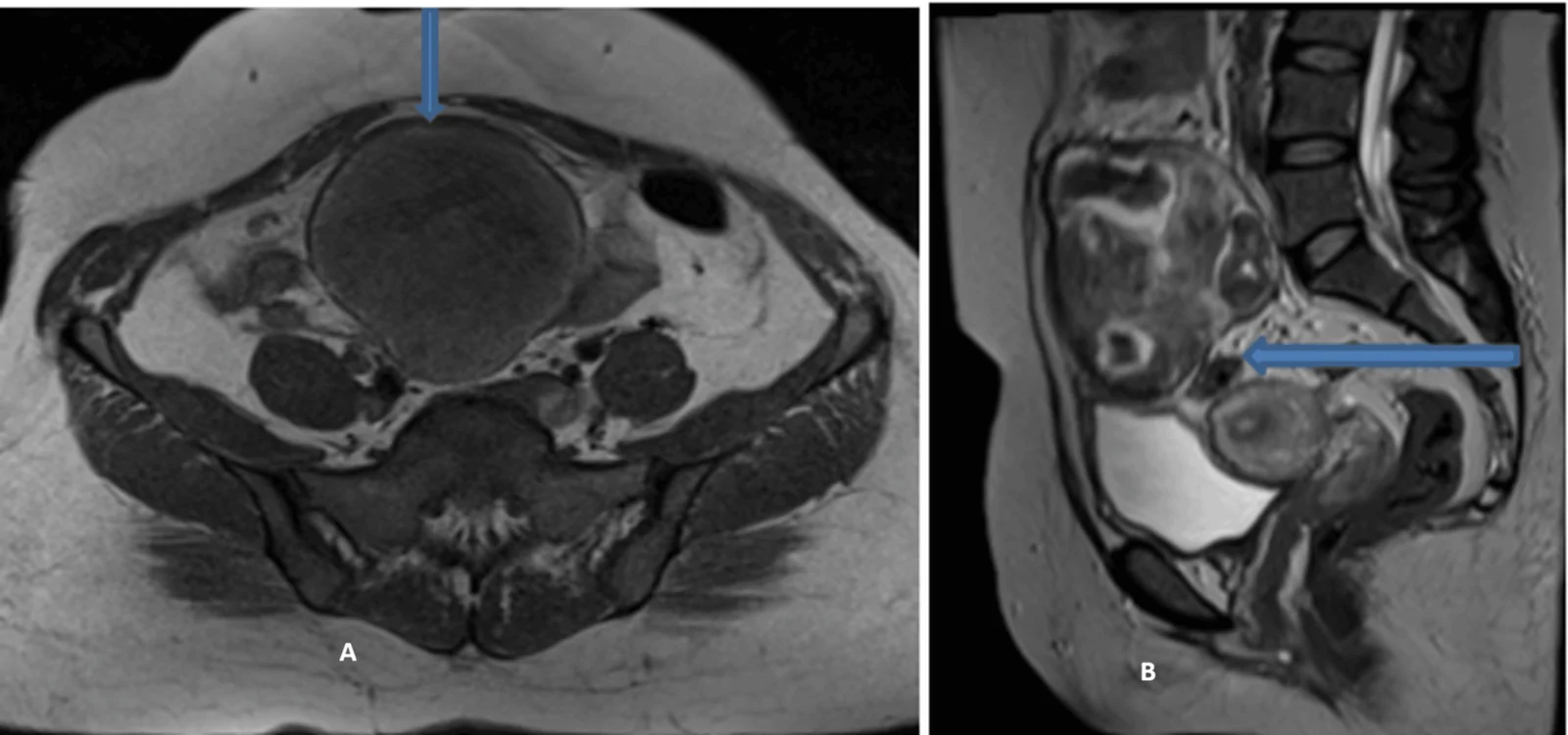
MRI pelvis Axial T1-weighted (A) and sagittal T2-weighted (B) images show a solid, well-defined, lobulated T1-hypointense and T2-heterogeneously hyperintense lesion with a T2-hypointense capsule. Small-bowel loops are seen between the lesion and the fundus of the uterus. MRI: magnetic resonance imaging

**Figure 5:**
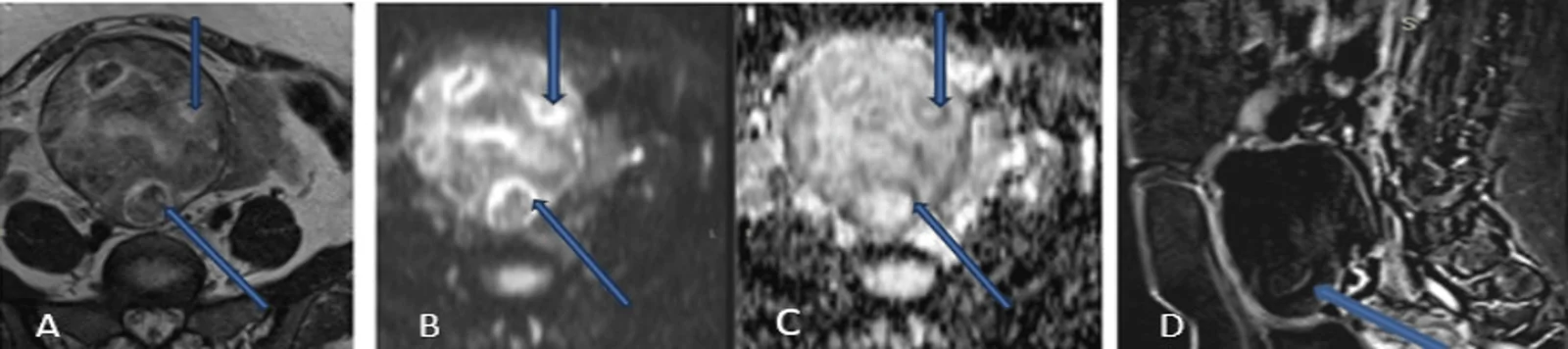
MRI pelvis Axial sections of the lesion on T2-weighted (A), DWI (B), and ADC (C) sequences, and a sagittal section on the T1-subtraction sequence (D), showed curvilinear areas of restricted diffusion (B, C) and enhancement (D), which corresponded to the T2-hyperintense areas (A). DWI: diffusion-weighted imaging, ADC: apparent diffusion coefficient, MRI: magnetic resonance imaging

**Figure 6:**
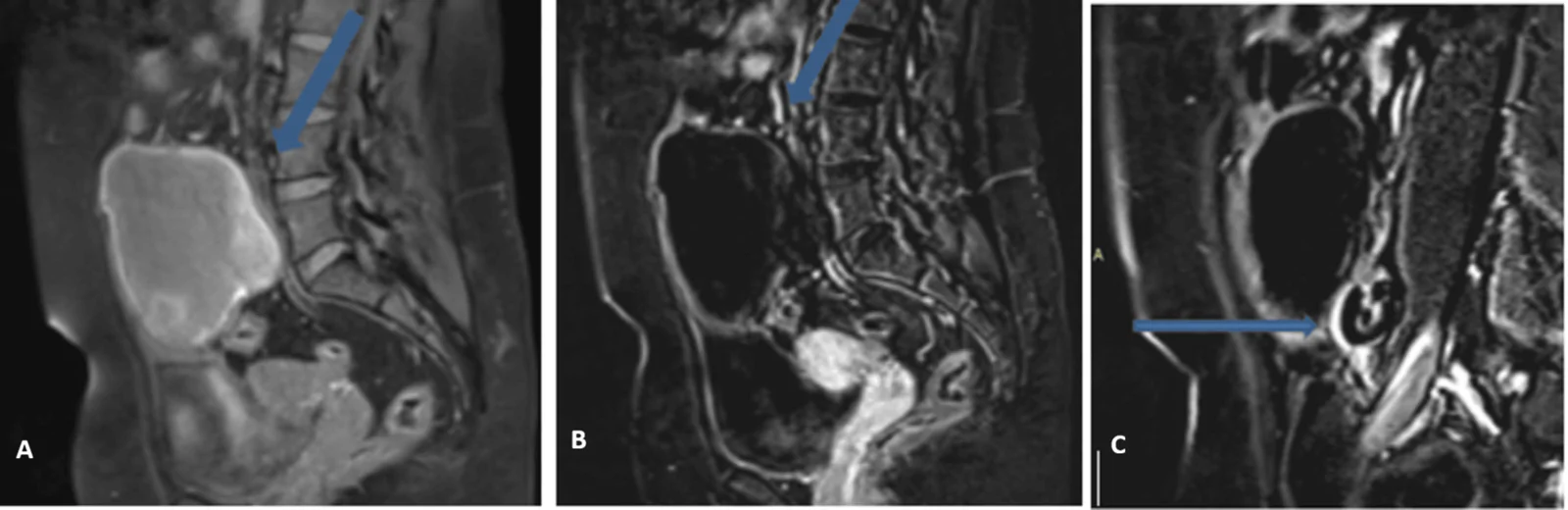
Contrast-enhanced MRI of the pelvis Sagittal sections show the lesion on T1 fat-saturated (A) and T1 post-contrast subtraction sequences (B, C). The lesion shows subtle peripheral enhancement (B) and is seen separately from the uterus, with intervening small-bowel loops (A). Serpiginous, tortuous, non-enhancing, tubular structures, likely representing thrombosed vessels, were seen in the right posterolateral aspect of the lesion, possibly representing a thrombosed vascular pedicle (C). MRI: magnetic resonance imaging

Treatment and operative findings

The patient underwent laparotomy with a midline abdominal incision, and the solid mass was excised. Intraoperative findings were those of a solid, intraperitoneal, hypogastric mass measuring 10 × 10 cm, adherent to the omentum and anterior parietal peritoneum. The lesion had multiple feeding vessels from the greater omentum and mesentery, with prominent, tortuous, thrombosed vessels in the right posterolateral aspect of the lesion while the remaining vasculature was intact (Figure 7A-7B). No uterine arterial supply was identified, and the uterus and ovaries were normal. At laparotomy, the absence of uterine arterial supply and the presence of multiple feeding vessels from the greater omentum and mesentery provided an anatomical explanation for the lesion's extrauterine location and vascularity. An intraoperative diagnosis of parasitic leiomyoma was made.

**Figure 7:**
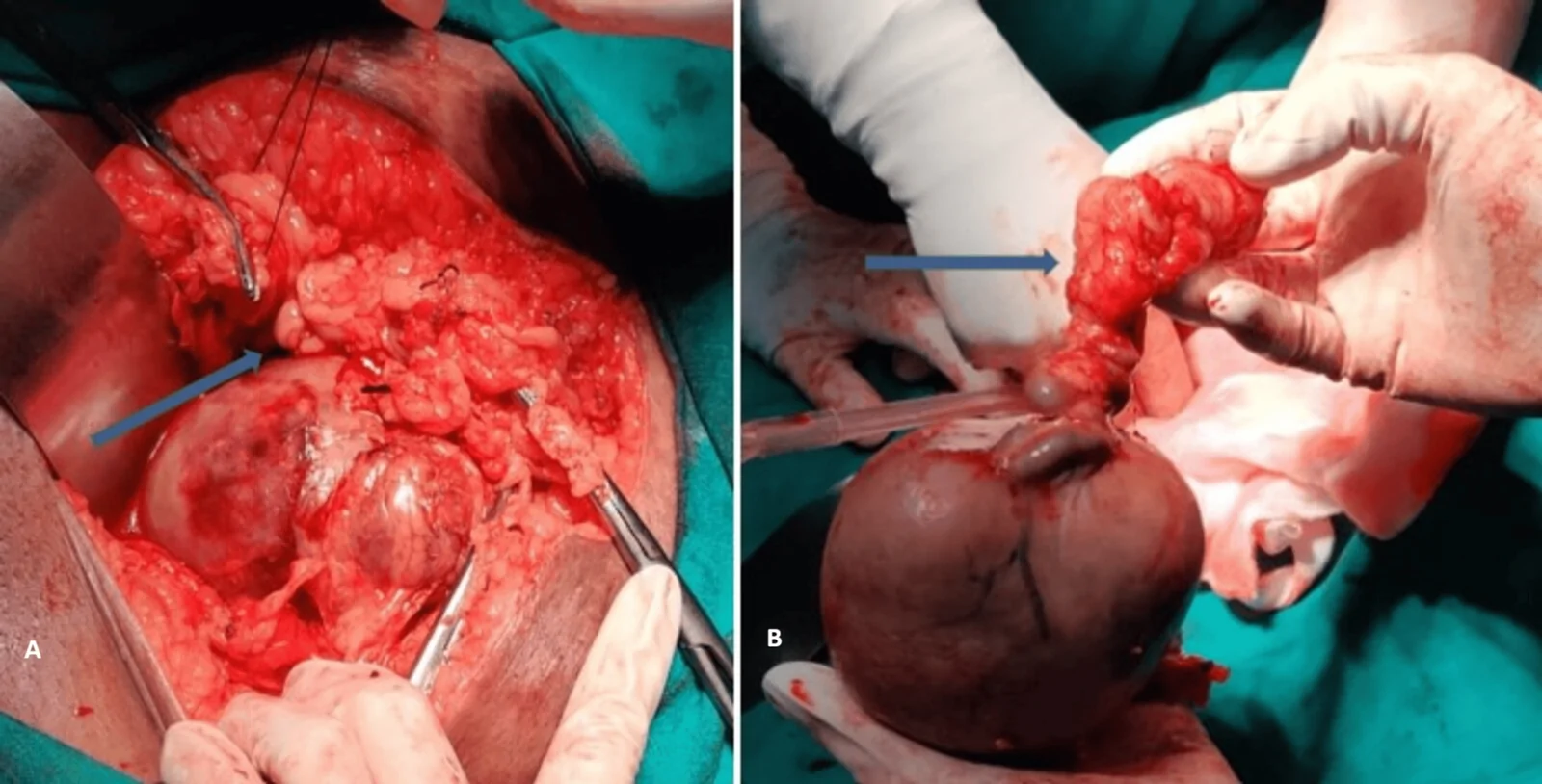
Intraoperative examination Solid, intraperitoneal, hypogastric mass adherent to the omentum and anterior parietal peritoneum (arrow; A). Excised, well-encapsulated, solid lesion with multiple feeding vessels and tortuous, thrombosed vessels (arrow) in the right posterolateral aspect of the lesion (B).

Follow-up and outcomes

A cut section of the specimen showed thrombosed feeding vessels (Figure 8A-8D) and a myxoid component and necrosis within the lesion (Figure 9A-9D). Histopathological examination showed features consistent with leiomyoma with extensive degenerative changes, hemorrhagic areas, and viable smooth muscle. The patient was discharged following the surgery.

**Figure 8:**
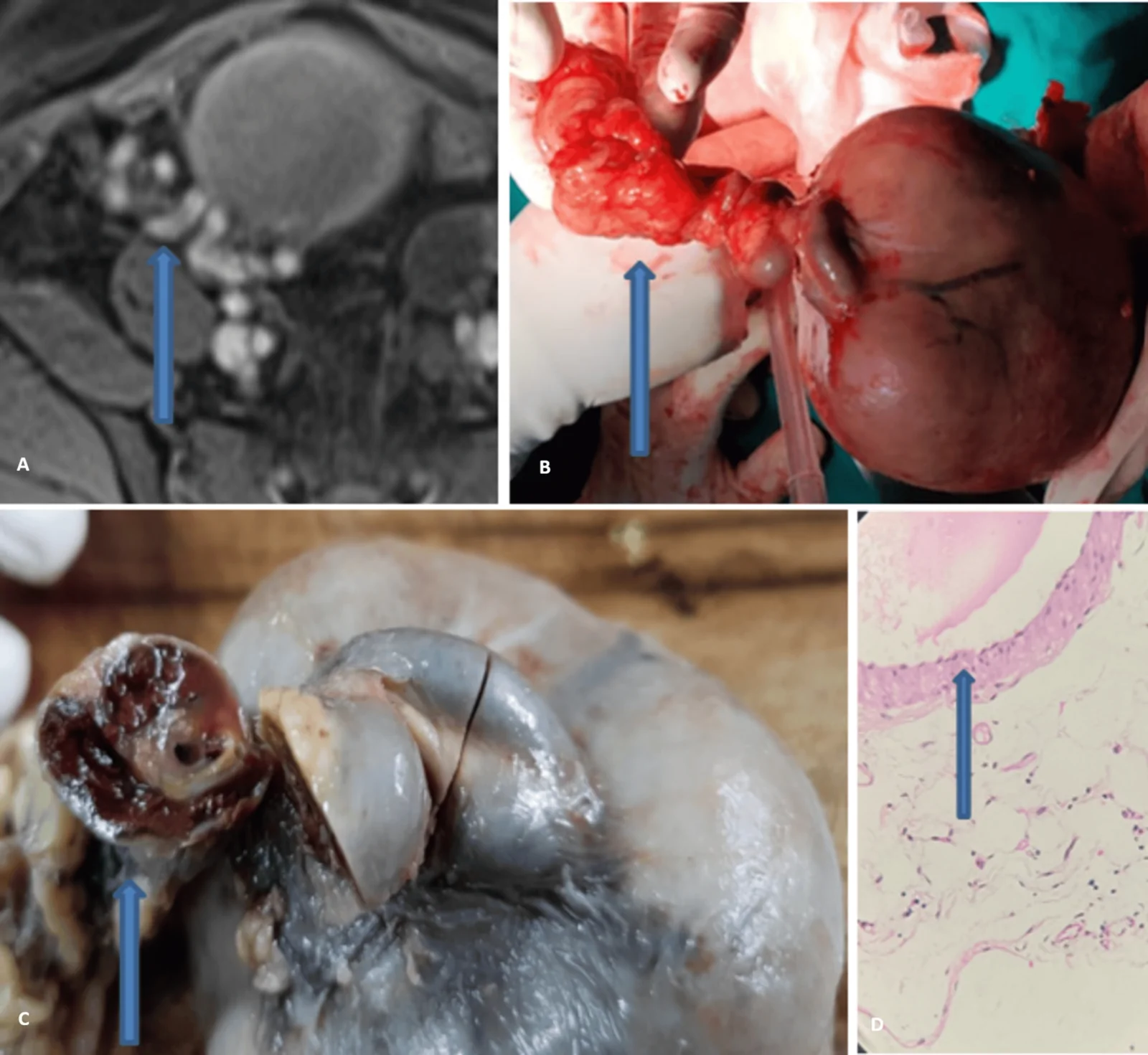
Clinico-radiopathological evaluation Pedicle with thrombosed vessels (solid blue arrow) on a T1 fat-saturated MRI sequence (A), surgical gross specimen (B), pathological gross specimen (C), and high-magnification photomicrograph (D). MRI: magnetic resonance imaging

**Figure 9:**
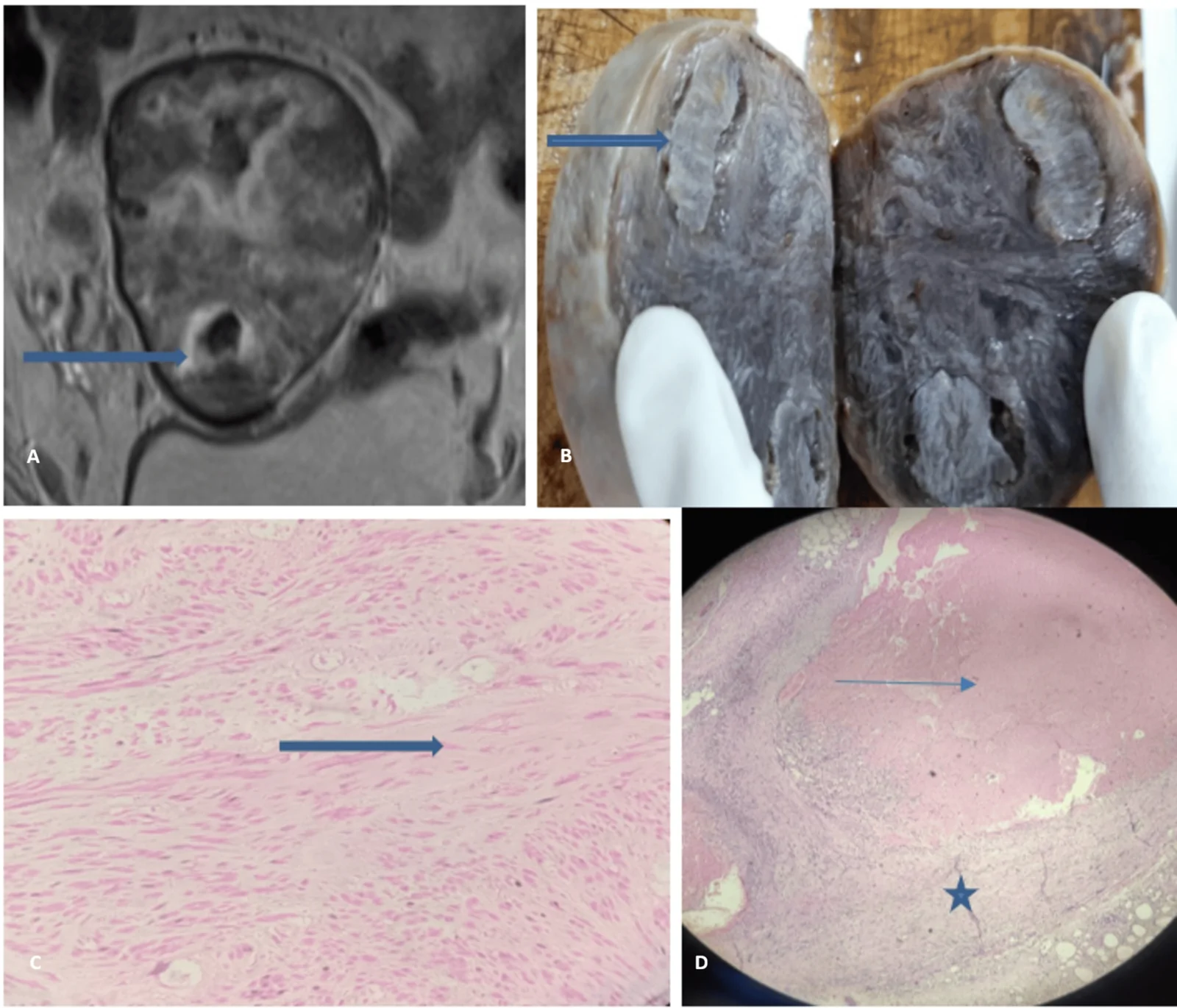
Clinico-radiopathological evaluation T1-weighted MRI sequence (A), pathological gross specimen (B), and high-magnification photomicrograph (C) showing areas of myxoid degeneration and necrosis, and low-magnification photomicrograph (D) showing smooth muscle bundles (blue star) in a whorled or fascicular pattern, separated by a hemorrhagic area and well-vascularized connective tissue (blue arrow). MRI: magnetic resonance imaging

Diagnostic dilemma

The clinico-radiopathological evaluation that followed sought to resolve the diagnostic dilemma. The differential diagnosis included mesenteric desmoid tumor, GIST, leiomyosarcoma, and ovarian neoplasms. Parasitic leiomyomas are typically well-circumscribed lesions with smooth muscle characteristics and an identifiable vascular supply. In contrast, desmoid tumors are often infiltrative and may be adherent to the anterior abdominal wall or mesentery [4,7]. The ultrasound finding of a well-defined, solid, heterogeneously hypoechoic lesion abutting the uterine fundus suggested a smooth muscle tumor; however, the absence of continuity with the uterus necessitated consideration of extrauterine lesions in the differential diagnosis.

GISTs usually arise from the gastrointestinal tract wall and can demonstrate heterogeneous enhancement, necrosis, and positivity for CD117 and DOG1 on immunohistochemistry, unlike leiomyomas [4]. Immunohistochemistry may be useful when the morphology is equivocal, particularly for distinguishing a smooth muscle tumor from GIST or other mesenchymal neoplasms. Histopathology revealed interlacing fascicles of benign spindle-shaped smooth muscle cells with cigar-shaped nuclei. Extensive areas of degeneration, hemorrhage, and necrosis were also noted, supporting the diagnosis of parasitic leiomyoma [4,7,8]. Although GIST was considered in the differential diagnosis given the intraperitoneal location, the operative findings and characteristic smooth muscle morphology on histopathological examination favored a parasitic leiomyoma. Mesenteric GIST was further excluded by histopathological evaluation, including Van Gieson staining. Van Gieson staining demonstrated the smooth-muscle-rich nature of the lesion and supported the histomorphological diagnosis of leiomyoma. Immunohistochemical evaluation was not performed in this case. Leiomyosarcomas commonly show irregular margins, invasive behavior, marked cytologic atypia, and increased mitotic activity on histopathology [7,8]. Ovarian neoplasms were also considered given the pelvic location of the mass; however, both ovaries were normal and visualized separately on imaging. In our case, the lesion was separate from the uterus and ovaries and demonstrated a thrombosed vascular pedicle with feeding vessels.

## Discussion

Parasitic leiomyoma is a rare type of extrauterine leiomyoma, presenting as a benign pelvic peritoneal smooth muscle mass distinct from the uterus, with a prevalence of 0.12% among leiomyomas [5,6].

Parasitic leiomyomas can be primary (detached, pedunculated subserous leiomyomas that have lost uterine vascularity) or iatrogenic (seeding of tissue fragments from previous myomectomy), the latter being the more common type [9,10]. Our case represents a primary parasitic leiomyoma, which is less common.

The majority occur in the pelvis (93%) [9,11]. The extra-pelvic location in our case is particularly uncommon. Extra-pelvic sites include the colon, omentum, small bowel, and the port site [12]. Recent literature indicates that most reported cases of parasitic leiomyoma are associated with coexisting uterine leiomyomas and a history of previous uterine surgery, particularly following laparoscopic morcellation [10]. In contrast, our patient had neither uterine leiomyomas nor any prior abdominal or pelvic surgery, making this an uncommon presentation.

Our patient was a premenopausal woman. In a review study by Ueda et al., women diagnosed with parasitic leiomyoma had a mean age of 40 years, with reported ages ranging from 18 to 79 years [13]. Most cases were identified among women of reproductive age, particularly those aged 18 to 49 years [14].

Fasih et al. reported that parasitic leiomyomas can present as extrauterine pelvic masses that compress the ureter, bladder neck, or urethra, potentially leading to urinary outflow obstruction of varying severity or secondary hydroureteronephrosis [3]. Our case had abdominal pain secondary to torsion of the parasitic leiomyoma and mild right hydroureteronephrosis but no urinary or bowel symptoms.

Leiomyomas typically demonstrate a distinct low signal intensity relative to the myometrium on T2-weighted imaging. These characteristic signal intensities are attributed to extensive hyalinization, which occurs in more than 60% of uterine leiomyomas [8].

Leiomyomas with myxoid degeneration demonstrate high signal intensity on T2-weighted MR imaging and show good enhancement, except in small areas corresponding to mucinous lakes or clefts. Hemorrhage and necrosis, apart from red degeneration, are uncommon but may occasionally be seen in leiomyomas.

Our lesion was predominantly hypointense on T1-weighted imaging and heterogeneously hyperintense on T2-weighted imaging due to myxoid degeneration, hemorrhage, and necrosis, which is uncommon.

## Conclusions

Parasitic leiomyoma is a rare but clinically significant entity that can pose a diagnostic challenge due to its atypical location and resemblance to other pelvic or intra-abdominal masses. Primary parasitic leiomyoma can present as an isolated intraperitoneal mass in the absence of previous pelvic surgery or a coexisting uterine leiomyoma. When evaluating an atypical pelvic or intra-abdominal mass, careful imaging assessment for features suggestive of an extrauterine leiomyoma may facilitate appropriate preoperative diagnosis and surgical planning. In this case, the thrombosed vascular pedicle, extrauterine feeding vessels, and extensive degenerative changes contributed to the diagnostic challenge and the atypical imaging appearance. Imaging provided important preoperative anatomical and vascular information, while a definitive diagnosis was established by correlating operative and histopathological findings.
